# A Neonate With Cytokine Storm Managed With Steroids, Therapeutic Plasma Exchange, and Tocilizumab

**DOI:** 10.7759/cureus.45138

**Published:** 2023-09-12

**Authors:** Amy LiKamWa, Kaitlin Kobaitri, Balagangadhar R Totapally

**Affiliations:** 1 Pediatrics, Herbert Wertheim College of Medicine, Miami, USA; 2 Critical Care Medicine, Nicklaus Children’s Hospital, Miami, USA

**Keywords:** steroids, interleukin-6, tocilizumab, therapeutic plasma exchange, hyperinflammation, cytokine storm, neonate

## Abstract

Neonatal cytokine storms, though rare, can induce hyperinflammation due to elevated interleukin-6 (IL-6), triggering multiorgan failure. We present the case of a term male neonate necessitating extracorporeal membrane oxygenation (ECMO) post-birth for persistent pulmonary hypertension due to meconium aspiration syndrome. Three days after weaning from ECMO support, steroids and therapeutic plasma exchange were initiated due to deteriorating thrombocytopenia, oxygenation, hemodynamic instability, and increased C-reactive protein (CRP) and ferritin levels. Elevated IL-6 prompted tocilizumab administration after four days of daily plasmapheresis. Post-tocilizumab infusion, notable enhancements in platelet counts, oxygenation indices, and CRP were observed, resulting in stable discharge of the child. Comprehensive evaluations for infections, including coronavirus disease 2019, as well as genetic and metabolic disorders, yielded negative results.

## Introduction

Cytokine storm in neonates is a known but uncommon condition. Reports of neonatal inflammatory conditions have increased during the coronavirus disease 2019 (COVID-19) pandemic [1]. An increase in interleukin-6 (IL-6) levels may lead to hyperinflammation in neonates leading to multiple organ failure [2]. Tocilizumab is a humanized monoclonal antibody that can prevent systemic inflammatory responses by blocking IL-6-mediated proinflammatory signaling. We report the case of a neonate with hyper IL-6 treated with steroids, therapeutic plasma exchange, and tocilizumab which led to a favorable outcome.

The details of the case were retrieved after Institutional Review Board approval (exempt study) for the study titled, *The use of tocilizumab in hospitalized children: a single pediatric center experience*. This case report has been accepted for presentation as a poster at the American Academy of Pediatrics National Conference and Exhibition, October 20-24, 2023, in Washington, DC.

## Case presentation

A male White Hispanic neonate born at 39 weeks gestation via elective C-section was transferred to the Pediatric Intensive Care Unit (PICU) for initiation of extracorporeal life support in the setting of persistent pulmonary hypertension, cardiogenic shock, transaminitis, and metabolic acidosis due to meconium aspiration syndrome. Maternal history was significant for positive group B streptococci and negative COVID-19. Upon arrival, the patient was started on extracorporeal membrane oxygenation (ECMO) support. He was placed on venoarterial (VA)-ECMO through jugular-carotid cannulation. When he was stable he was transitioned to conventional mechanical ventilation after 12 days of ECMO run. The patient was noted to be large for gestational age (4.7 kg) with ruddy, plethoric skin, and a mildly distended abdomen. He required continuous monitoring and high-level supportive care, including extracorporeal life support to maintain organ failure and support vital physiological requirements, invasive mechanical ventilation for respiratory failure, continuous vasoactive infusions to support blood pressure, and continuous benzodiazepine and opiate infusions for sedation and analgesia.

**Figure 1:**
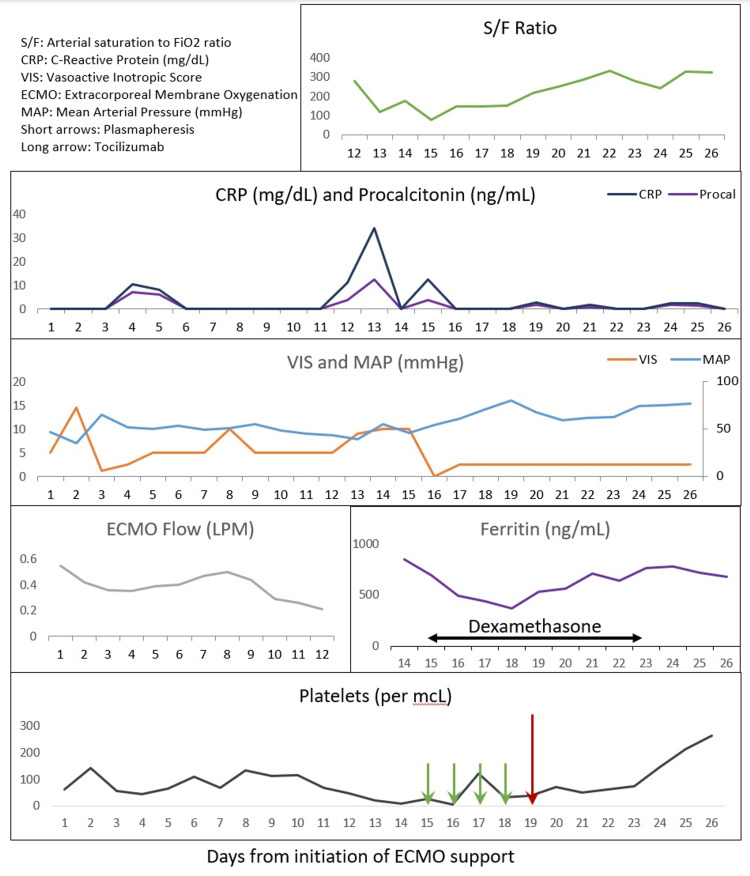
The timeline of cardiorespiratory support, laboratory data, and therapies in a neonate with cytokine storm.

Due to worsening thrombocytopenia, oxygenation (decreasing S/F ratio), hemodynamic instability, and elevated C-reactive protein (CRP) and ferritin (851 ng/mL) levels, steroids and therapeutic plasma exchange were initiated three days after the ECMO support was discontinued (Figure 1). As the patient was showing signs of severe inflammation, including IL-6 levels of >400 pg/mL (normal: ≤1.8 pg/mL), 56 mg of tocilizumab was administered intravenously after four days of therapeutic plasma exchange. Genetic and metabolic testing were normal. COVID-19 polymerase chain reaction (PCR) testing was negative. IL-1 alpha level was below the detection limit of 3.9 pg/mL, IL-1 beta level was 4.0 pg/mL (normal: <1.0 pg/mL), tumor necrosis factor level was 3.4 pg/mL (normal: <2.8 pg/mL), soluble IL-1 receptor assay was 2,055 U/mL (normal: 398-1,940 U/mL), natural killer cell function was normal, and testing for heparin-induced thrombocytopenia was negative. Viral studies for respiratory viruses, adenovirus, enterovirus, and Epstein-Barr virus were negative. Microbiological cultures and Karius® non-invasive blood test (Karius®, Redwood City, CA, USA) were negative. Fungitell® (Fungitell®, East Falmouth, MA, USA) and aspergillus PCR were negative. He was treated with multiple courses of antibiotics and antifungals during hospitalization for presumed sepsis. Following the infusion of tocilizumab, there were marked improvements in platelet levels, S/F ratio, and CRP. He received 27 platelet transfusions during 12 days of ECMO and 16 platelet transfusions for seven days after ECMO. The infant did not require any platelet transfusion one day after tocilizumab infusion. The patient was discharged home 88 days later in stable condition with a gastrostomy tube. Since discharge, the gastrostomy tube has been discontinued and he is progressing well with some lag in achieving the developmental milestones.

## Discussion

We present a neonate who developed thrombocytopenia, worsening organ failure, and hyperinflammation and improved clinically with steroids, therapeutic plasma exchange, and tocilizumab. The findings of elevated inflammatory markers and IL-6 levels, thrombocytopenia, and multiple organ failure in this infant are consistent with cytokine storm. Cytokine storm or cytokine release syndrome is a dysregulated response to infectious and non-infectious stimuli with cytokine release and inflammation [3]. Cytokine storm refers to a group of immune dysregulation disorders where the body experiences systemic inflammation, constitutional symptoms, and multiorgan dysfunction. If not properly treated, it can progress to multiorgan failure [3]. Elevated IL-6 levels are found in several cytokine storm disorders. Glucocorticoids, monoclonal antibodies against several cytokines, and sirolimus are successfully used in various types of cytokine storms [3]. Therapeutic plasma exchange is a known therapy in patients with thrombocytopenia-associated multiple organ failure from sepsis [4].

IL-6 elevation activates the nuclear factor kappa B (NF-κB) and Janus kinase/signal transducer and activator of transcription (JAK/STAT3) pathways. The hyperactivation of the NF-κB and STAT3 inflammatory pathways induces the IL-6 amplifier leading to an enhanced NF-κB activation [5]. The overactivation of NF-κB results in cytokine storm leading to acute respiratory distress syndrome, multiorgan failure, and coagulation dysfunction. Steroids act at various stages of the inflammatory cascade. The anti-inflammatory effects of glucocorticoids primarily stem from their ability to directly influence gene transcription. This involves the regulation of specific genes responsible for controlling cell activation and the production of inflammatory mediators. They inhibit prostaglandins and leukotrienes, pro-inflammatory mediators that contribute to the dilation of blood vessels, increase vascular permeability, and attract immune cells to the site of inflammation. They also block the activity of phospholipase A2, an enzyme involved in the production of arachidonic acid which is the precursor for prostaglandins and leukotrienes. Steroids also suppress the production of proinflammatory cytokines, such as ILs and tumor necrosis factor. Additionally, they stabilize cell membranes, reducing permeability and preventing the release of inflammatory substances from immune cells. The overall effect of steroids is to suppress the immune response and reduce inflammation [6]. Steroids have been used in various inflammatory conditions, including in the management of cytokine storms. Dexamethasone is a recommended treatment for COVID-19-associated cytokine storm [7]. Dexamethasone is used in the management of hemophagocytic lymphohistiocytosis [8].

The guidelines for therapeutic plasma exchange have been published by the American Society of Apheresis [9]. Therapeutic plasma exchange is an accepted therapy for thrombocytopenia-associated multiple organ failure in children, mainly considered when there is evidence of immune-mediated damage to organs [4]. Exchanging the patient’s plasma with donor plasma removes inflammatory mediators from the patient’s plasma that might be contributing to organ dysfunction [10]. Although therapeutic plasma exchange is an uncommon procedure in neonates and is fraught with technical challenges, it has been successfully performed in neonates [11,12]. We exchanged the venous line of VA-ECMO during decannulation and used it for the therapeutic plasma exchange procedure. The challenges with vascular access and complications from the procedure should be considered for using the therapeutic plasma exchange procedure.

IL-6 level was measured before starting the therapeutic plasma exchange and was found to be elevated in our case which prompted us to administer a dose of tocilizumab. IL-6 is a known mediator of inflammation in neonates. It is known to be elevated in early and late-onset neonatal sepsis as well as in fetal inflammatory response syndrome [2] Hyperinflammation may occur due to sepsis, an autoimmune condition, or an innate response. Tocilizumab, an IL-6 receptor blocker, is approved for many conditions with elevated IL-6 levels [13]. It has been used in hyperinflammation associated with COVID-19 [14,15]. It is primarily used in the treatment of rheumatoid arthritis, juvenile idiopathic arthritis, giant cell arteritis, and cytokine release syndrome. As one possible concern with the use of tocilizumab is its immunosuppressive effects, the risks of infection must be monitored [13,16].

COVID-19-associated multisystem inflammatory syndrome in neonates, similar to that seen in children, has been described which can present with thrombocytopenia and multiple organ dysfunction [17]. We ruled out active infection leading to hyperinflammation or any evidence of maternal or post-natal severe acute respiratory syndrome coronavirus 2 infection in this neonate. The use of ECMO support itself can lead to inflammation. Extensive workup for infectious causes and genetic predisposition for hyperinflammation were negative in this child. The initiation of ECMO brings an immediate and complex inflammatory reaction in patients, as seen in systemic inflammatory response syndrome, which can lead to the widespread activation of the endothelium and induction of proinflammatory cytokine secretion [18].

We simultaneously used three immunomodulating therapies, namely, dexamethasone, therapeutic plasma exchange, and tocilizumab, in our patient. We cannot be certain which one or more of the therapies were responsible for the improvement in the patient’s clinical condition. Temporally, there was an immediate improvement in platelet count after the administration of tocilizumab. In addition, we are not certain of the inciting stimulus for cytokine storm in our newborn.

## Conclusions

This case underscores the importance of investigating hyperinflammatory conditions in neonates experiencing multiorgan failure unrelated to sepsis. When managing such cases, potential therapeutic strategies including steroids, therapeutic plasma exchange, and cytokine-modulating drugs should be contemplated and tailored to the specific cause and cytokine pattern. For neonates caught in a cytokine storm, these interventions hold the potential to be lifesaving.
